# A comparison of physicians and medical assistants in interpreting verbal autopsy interviews for allocating cause of neonatal death in Matlab, Bangladesh: can medical assistants be considered an alternative to physicians?

**DOI:** 10.1186/1478-7954-8-23

**Published:** 2010-08-17

**Authors:** Hafizur R Chowdhury, Sandra C Thompson, Mohammed Ali, Nurul Alam, Mohammed Yunus, Peter K Streatfield

**Affiliations:** 1 Centre for International Health, Curtin Health Innovation Research Institute, Curtin University of Technology, GPO Box U1987, Perth, Western Australia 6845, Australia; 2 Matlab Health Research Centre, International Centre for Diarrhoeal Disease, Research, Bangladesh (ICDDR,B), GPO Box 128, Dhaka 1000, Bangladesh; 3 Combined Universities Centre for Rural Health, University of Western Australia, PO Box 109, Geraldton, Western Australia 6531; 4 Health and Demographic Surveillance Unit, Public Health Sciences Division, International Centre for Diarrhoeal Disease Research, Bangladesh (ICDDR,B), GPO Box 128, Dhaka 1000, Bangladesh; 5 Public Health Sciences Division, International Centre for Diarrhoeal Disease, Research, Bangladesh (ICDDR,B), GPO Box 128, Dhaka 1000, Bangladesh

## Abstract

**Objective:**

This study assessed the agreement between medical physicians in their interpretation of verbal autopsy (VA) interview data for identifying causes of neonatal deaths in rural Bangladesh.

**Methods:**

The study was carried out in Matlab, a rural sub-district in eastern Bangladesh. Trained persons conducted the VA interview with the mother or another family member at the home of the deceased. Three physicians and a medical assistant independently reviewed the VA interviews to assign causes of death using the International Classification of Diseases - Tenth Revision (ICD-10) codes. A physician assigned cause was decided when at least two physicians agreed on a cause of death. Cause-specific mortality fraction (CSMF), kappa (k) statistic, sensitivity, specificity, and positive predictive values were applied to compare agreement between the reviewers.

**Results:**

Of the 365 neonatal deaths reviewed, agreement on a direct cause of death was reached by at least two physicians in 339 (93%) of cases. Physician and medical assistant reviews of causes of death demonstrated the following levels of diagnostic agreement for the main causes of deaths: for birth asphyxia the sensitivity was 84%, specificity 93%, and kappa 0.77. For prematurity/low birth weight, the sensitivity, specificity, and kappa statistics were, respectively, 53%, 96%, and 0.55, for sepsis/meningitis they were 48%, 98%, and 0.53, and for pneumonia they were 75%, 94%, and 0.51.

**Conclusion:**

This study revealed a moderate to strong agreement between physician- assigned and medical assistant- assigned major causes of neonatal death. A well-trained medical assistant could be considered an alternative for assigning major causes of neonatal deaths in rural Bangladesh and in similar settings where physicians are scarce and their time costs more. A validation study with medically confirmed diagnosis will improve the performance of VA for assigning cause of neonatal death.

## Introduction

Accurate information on cause of death in communities is important for effective planning and evaluation of various health care interventions [1,2]. Deaths occurring during the first four weeks of life, the neonatal period, are particularly important because they account for around two thirds (63%) of all infant deaths and nearly half (47%) of under-five child deaths in Bangladesh [3] and 42% globally [4]. As post neonatal and child mortality continue to decline due to successful implementation of proven interventions [5], the relative contribution of neonatal death to child deaths is increasing. Therefore, collection of accurate information on neonatal events leading to death is critical.

Of 4 million annual neonatal deaths, 99% occur in low- and middle-income countries [1,6]. As the great majority of births and neonatal deaths in developing countries occur at home outside the formal health care setting, it is difficult to ascertain the cause of death. In such situations, verbal autopsy (VA) can be a valuable, low-cost and practical tool to ascertain cause of death.VA is a practical tool for assigning a probable cause of death where routine death registration is non-existent or inadequate and autopsies are rarely available. The VA methodology utilizes retrospective information collected during interviews with care givers or family members about the symptoms, signs, care seeking and other events related to the deceased and their illness or circumstances prior to death [7,8]. This information is summarized or reviewed to give a most likely cause of death. However, the method used to assign cause of death from the information obtained by VA should be appropriate, valid, reliable and inexpensive.

There are a number of methods for interpreting VA interviews to arrive at a cause of death. These include review by a medically qualified person such as a physician, use of algorithms, neural networks or a probabilistic approach [9]. Each method has its advantages and disadvantages in terms of validity, cost effectiveness, complexity of technique and repeatability [10,11]. Review by a physician is considered to be reasonably accurate owing to their professional training and knowledge about the disease pattern of the country, and not reliant upon complex computer algorithms that would need to be adjusted for local conditions. Thus, they could provide robust estimates of cause-specific mortality fractions (CSM) for common illnesses. Physician review for interpreting VA interviews requires a considerable amount of scarce physician time. Algorithms and neural networks have been explored with inconclusive results in terms of validity [11]. Algorithms work well for identifying distinctive and memorable clinical syndromes such as neonatal tetanus, measles and injury, but work inconclusively for pneumonia and malaria [12,13]. Recently, a new probabilistic approach called interVA has been evaluated with physician review and was found to have high consistency between physician- and the model- assigned cause of death as well as being reliable and cost-effective [14].

Usually, physician review and algorithms are used after validation with hospital diagnosis as the gold standard. But the reliability and validity of cause of death are dependent on the quality of information obtained and "gold standard" diagnoses. Hospital diagnosis has problems as a reference standard since these diagnoses are often not representative of community morbidity. Moreover, in resource-constrained health care settings, hospital diagnoses are often unavailable, and even when available, are limited by inadequate clinical data and record keeping. In addition, use of varied methods and instruments in VA assessment limits the comparability of findings across settings and over time [10,15].

While review by a physician - often multiple physicians - for allocating cause of death is the most commonly used method, this process requires a considerable amount of scarce physician time which is not practical in many low-income developing countries. Studies have shown that non-physician and mid-level providers are able to conduct specified clinical tasks with adequate training [16,17]. A recent study also showed that, after training the mid-level workers (nurses and midwives) achieved a level of both cognitive and applied knowledge comparable to physicians to allocate cause of death using VA data [18].

The use of a medical assistant who has three years of institutional medical training has been considered as an alternative to physician review for VA data. Both physician and medical assistant would be expected to have similar cognitive approaches to assigning cause of death. However, to date no research has been conducted to evaluate the performance of medical assistants in this regard.

The International Centre for Diarrhoeal Disease Research, Bangladesh (ICDDR,B) maintains a comprehensive Health and Demographic Surveillance System (HDSS) in a rural area in Bangladesh. Since 2003, the HDSS has been using a structured VA questionnaire. This paper reports the diagnostic agreement between medical assistants and physicians in assigning cause of death by reviewing VA interview data in the Matlab HDSS during the period 2003-2004 [19].

## Methodology

### Study design and population

The study was conducted in Matlab, a rural sub-district in eastern Bangladesh, where ICDDR, B maintains a longitudinal health and demographic surveillance system covering a population of ~ 224,000 people [19]. Community Health Research Workers (CHRW), who are literate married women that were recruited from among local residents, carry out monthly household visits to collect information on demographic events (birth, death, migration, abortion, marriage, divorce, etc) using pre-coded colored forms to record the events. Living in the village and having a limited number of households to survey, they are unlikely to miss any vital events. These data are collated and maintained in the HDSS databases [19]. This study investigated and analyzed all 365 neonatal deaths that occurred in 2003 and 2004 in the Matlab area under HDSS.

### Verbal autopsy

This questionnaire was developed by the Verbal Autopsy Working Group of the International Network of Field Sites with Continuous Demographic Evaluation of Populations and their Health in Developing Countries (INDEPTH; see http://www.indepth-network.org), which is based on the World Health Organization VA questionnaire. The questionnaire was adapted to local customs and culture and translated in Bangla by the VA team at the Matlab HDSS in 2003. It includes both open-ended and close-ended questions on the pregnancy, birth, and illnesses leading up to the neonatal death, as well as information on health care-seeking behaviour during the fatal illness episode.

### Data collection

The CHRW identified the deaths and filled up a registration slip (date of death, identification number, etc) during their monthly house- to- house visit which was sent to the block supervisor. These were then uploaded to the HDSS records. An interviewer trained in verbal autopsy visited the home 2 to 6 weeks after the date of death to conduct the interview. After obtaining informed verbal consent, interviews were conducted, generally with the mother, but sometimes with other family members to supplement the interview data. Descriptive statements were recorded in the open part of the questionnaire in Bangla, preserving local idioms and refraining from any alteration or translation. Interviews generally lasted for 40 to 60 minutes depending on the illness history and the emotional state of the caretakers.

### Cause of death assignment

All deaths were independently reviewed by three physicians and a medical assistant to assign a direct cause of death and an originating/underlying cause of death when possible. Physicians were general doctors from ICDDR, B Matlab Hospital and were aware of the Integrated Management of Childhood Illness (IMCI) guidelines and booklets. The medical assistant was a paramedic with three years of institutional medical training who was employed at the HDSS project. The three physicians were provided with two interactive orientation sessions, each with a two-hour duration, regarding the concept of VA and how to review the VA questionnaire to assign cause of death and how to identify ICD-10 codes using the ICD-10 manual. The medical assistant was also provided the same orientation, but in separate sessions. None of the physicians had previous experience with VA, while the medical assistant had experience with VA in the demographic surveillance program at Matlab. Their role was to review all sections of the questionnaire including health care seeking and to assign direct causes of death and originating cause of death when possible using ICD-10 codes. Of the three physicians involved in the review process for assigning cause of death, at least two had to agree on the cause of death which could be used as the reference diagnosis for assessing the reliability and agreement of performance by the medical assistant (MA).

The process of identification of physician assigned causes was done in three steps. First, all 365 cases were reviewed for their direct causes of death. Of these, in 152 cases all three physicians agreed on the cause of death. In another 174 cases, two physicians agreed on the direct cause of death. This left 39 cases where there was no physician agreement. In these 39 cases, the direct and originating/underlying causes of death were reviewed to check whether there could be agreement between any of the physician- assigned direct cause with originating cause of any of the other two physicians. Thirteen such cases were found. This process increased the number of agreement among physician- assigned direct causes of death but still left 26 cases where there was no agreement between direct and originating causes of the three physicians.

### Data analysis

All data were entered via Visual FoxPro data entry screen into the Oracle database. Data was analyzed with Stata, version 9. Cause of death was grouped into categories when appropriate. A direct cause was defined as a disease or condition that directly led to the death of the neonate. Cause-specific mortality fraction (CSMF), kappa score, sensitivity, specificity, and positive predictive values were used for assessing the reliability and diagnostic agreement of diagnoses assigned by the MA. The following kappa (k) scale was used to rate the strength of agreement: a k < 0.21 was considered poor, a k between 0.21 and 0.40 fair, a k between 0.41 and 0.60 moderate, a k between 0.61 and 0.80 good, and a k > 0.80 very good [20]. There are no rule of thumb criteria to evaluate the agreement of VA technique for assigning causes of death. A recent study in Tanzania used criteria such as sensitivity >50% and specificity >1-CSMF as the gold standard, which did not allow a high degree of misclassification. Specificity is more important for validity assessment when CSMF is low [21].

### Ethical approval

The study interviewers obtained verbal consent from all participants. The Human Research Ethics Committee of Curtin University and the Ethical Review Committee of ICDDR, B approved the study.

## Results

Inter-rater variation, the MA versus each individual physician, is an important criterion for judging the quality of the method for assigning cause of death, and Table 1 shows the variation among the physicians. There was agreement by at least two physicians in 93% of cases leaving only 7% of cases where there was no agreement between any two of the physicians.

**Table 1:** Agreement between physicians interpreting the same VA reports

	Neonatal deaths (N = 365)	
	
Level of physician agreement	n	%
No agreement	26	(7.1)
Two physicians agreed on a cause	187	(51.2)
Three physicians agreed on a cause	152	(41.6)
		
At least two physicians agreed	339	(92.9)

*Total deaths = 365*		

Inter- method, single MA versus a panel of physicians, and inter-rater variations (percent agreement) are important in evaluating a method for its routine use for assigning causes of death. In the great majority of cases (81.6%), there was agreement between the MA and at least one physician on a cause of death (Table 2). The agreement in assigning causes between the MA and any particular physician varied, with 61% agreement with physician1, 67% with physician2 and 54% with physician3. There was no agreement between the MA and any physician in 18% of cases. Table 3 compares cause-specific mortality fractions (CSMF) between individual physicians and the MA, which show that birth asphyxia was the most frequently assigned cause by all the assessors, and only physician 3 differed somewhat from the MA and the other two physicians with respect to the other four major causes. Table 4 presents the kappa score for agreement between the individual physicians themselves, and Table 5 shows almost similar variation like variation between individual physician and the MA.

**Table 2:** Agreement between the MA and three physicians interpreting the same VA reports

	**deaths = 365**	
	
**Agreement between MA and physicians**	**n**	**%**
	
MA agreed with physician1	223	(61.1)
MA agreed with physician2	243	(66.6)
MA agreed with physician3	198	(54.3)
		
No agreement between MA and physicians	67	(18.4)

**Table 3:** Cause-specific mortality fraction of physician and medical assistant- assigned direct cause of death (N = 365)

	MA		Physician1		Physician2		Physician3	
				
Cause of death	n	(%)	n	%	n	%	n	%
Prematurity/Low Birth weight(LBW)	41	(11.2)	64	(17.5)	59	(16.2)	29	(7.9)
Birth asphyxia	151	(41.4)	151	(41.4)	141	(38.6)	194	(53.2)
Respiratory Distress Syndrome (RDS)	39	(10.7)	23	(6.3)	49	(13.4)	13	(3.6)
Pneumonia	35	(9.6)	22	(6.0)	26	(7.1)	13	(3.6)
Sepsis/Meningitis	29	(7.9)	57	(15.6)	33	(9.0)	72	(19.7)

**Table 4:** Kappa score between physicians (two raters) in assigning major cause of cause of death

Cause of death	Physician1 and 2	Physician1 and 3	Physician 2 and 3
	**Kappa Score**		
	
Prematurity/LBW	0.46	0.31	0.26
Birth asphyxia	0.69	0.68	0.63
RDS	0.51	0.19	0.25
Pneumonia	0.60	0.25	0.38
Sepsis/Meningitis	0.50	0.52	0.42

**Table 5:** Agreement between physicians and the medical assistant for assigning major direct causes of death

	Physician1 & MA		Physician2 & MA		Physician3 & MA	
	
*Cause of death*	*PA*	*kappa*	*PA*	*kappa*	*PA*	*Kappa*
Birth asphyxia	89.6	0.79	87.9	0.75	81	0.63
Prematurity/LBW weight	87.7	0.50	90.1	0.58	87.9	0.31
RDS	91.8	0.47	93.4	0.69	90.1	0.27
Pneumonia	92	0.45	94.2	0.63	91.8	0.34
Sepsis/Meningitis	86.5	0.36	92.9	0.54	86.3	0.44

### Reliability (kappa score) between individual physicians & the medical assistant (inter-rater)

The percent agreement (PA) for the physicians and the MA methods (Table 5) were reasonably high for the five major causes of death (81% to 94%). The corresponding kappa values ranged from moderate to good for all of the agreements between the MA and physician1 and physician2 except, for sepsis/meningitis (0.36) between the MA and physician1. The degree of agreement between the MA and physician3 varied and the corresponding kappa values indicated a good agreement for birth asphyxia (0.63), moderate agreement for sepsis/meningitis (0.44), and a fair level of agreement (0.27-0.31) for the three other major categories of death.

### Major cause-specific mortality fraction of methods

A summary of major causes of death by method for assigning cause of death for physicians (at least two physicians agreeing on a cause) compared to the MA (Table 6) shows that the MA assigned a slightly smaller proportion of deaths to birth asphyxia (41.5% MA versus 44.9% physicians). For prematurity/low birth weight, the MA assigned a smaller proportion of deaths (11.3%), compared to the physician method (15.1%). There were small variations for the other causes of deaths as well, such as sepsis/meningitis, Respiratory Distress Syndrome (RDS) and pneumonia. The five major causes of death accounted for more than 80% of neonatal deaths both in physician and medical assistant assigned causes of deaths

**Table 6:** Cause-specific mortality fraction of physician (at least two physicians agreed) and medical assistant-assigned direct cause of death

	Physician		MA	
		
Cause of death	N	%	N	%
		
Birth asphyxia	164	(44.9)	151	(41.5)
Prematurity/LBW Weight	55	(15.1)	41	(11.3)
Sepsis/Meningitis	45	(12.3)	29	(8.0)
RDS	25	(6.9)	39	(10.7)
Pneumonia	20	(5.5)	35	(9.6)
Others				
Congenital anomaly	3	(0.8)	10	(2.8)
Specified rare^a^	22	(6.0)	57	(15.7)
Undetermined^b^	26	(7.1)	n/a	n/a
Unspecified cause	5	(1.4)	2	(0.6)
Total	365	(100)	364	(100)

^a ^Causes with small numbers are grouped under other specified

^b ^Cause where three physicians' differed on a cause of death

### Reliability and diagnostic agreement of MA performance compared to two physician agreed major causes of death (Inter-method)

Agreement between the MA and the physicians for major causes of death was good for birth asphyxia and moderate for prematurity/low birth weight; RDS, pneumonia and meningitis/sepsis (Table 7).

**Table 7:** Summary diagnostic agreement and reliability of the medical assistant approach compared to physician (at least two physicians agreeing on a cause) for assigning major direct causes of death

		Diagnostic agreement			Reliability	
			
Condition	Review Method	Sensitivity (%)	Specificity (%)	PPV (%)	Kappa	Strength of agreement
Prematurity/LBW	MA	52.7	96.1	70.7	0.55	Moderate
						
Birth asphyxia	MA	83.5	93.0	90.7	0.77	Good
						
RDS	MA	80.0	94.4	51.3	0.59	Moderate
						
Pneumonia	MA	75.0	94.2	42.9	0.51	Moderate
						
Sepsis/Meningitis	MA	47.7	97.5	72.4	0.53	Moderate

For agreement assessment, considering physician as reference category, MA had a high level of sensitivity (>75%) and specificity (>90%) for assigning the major cause of death except for sepsis/meningitis and prematurity/low birthweight where the sensitivity was 48% and 53% respectively. MA also had a high level of PPV (>70%) for assigning the same cause of death as the physicians for birth asphyxia, prematurity/low birth weight, and sepsis/meningitis, although the PPV for pneumonia and RDS were a bit low (43-51%) (Table 7)

## Discussion

Comparison of the physicians' review and the medical assistant's review for determining major direct causes of death showed that the two approaches were reasonably consistent for the cause-specific mortality fraction (CSMF) of the major neonatal causes of death in this Matlab community setting. Patterns of major causes were also similar with those reported from other community-based studies in South Asian countries [3,22-25]. Five major categories of neonatal deaths accounted for more than 80% of all neonatal deaths, as determined by the two out of three physician agreement "gold standard" and the MA. However, there was some variation with regards to rank of individual causes. These five major causes were birth asphyxia, prematurity/low birth weight, respiratory distress syndrome, pneumonia and sepsis/meningitis.

In developing countries, cause and information on death certificates can often be inadequate and not always in agreement with clinical records review, as demonstrated in a South African study. The study concluded that combined clinical records and VA can provide more complete information than death certificate alone in areas with poor quality of mortality data [26].

The VA puts emphasis on causes of death which have major public health importance, and relies less on individual causes of death [14]. This public health or community approach has been reflected here by the two VA approaches showing common patterns of causes of neonatal death reported earlier from the developing countries. Therefore, it seems logical to propose that an MA could be used for allocating major causes of public health importance where physician time is scarce.

Our study found a good level of agreement between physician and MA review when assigning birth asphyxia as a cause of death (k = 0.77). A kappa value of 0.80 indicates almost perfect agreement between two methods. The corresponding agreement was moderate for sepsis/meningitis (k = 0.53), prematurity/low weight (k = 0.55), RDS (k = 0.59), pneumonia (k = 0.51) and meningitis/sepsis (k = 0.53).

There are no published data comparing physician review with an MA's review. However, a study from rural Nepal comparing physician review with algorithm-based diagnoses in verbal autopsies for neonatal deaths provides interesting information about the pattern of causes allocated by physicians. In the Nepalese study, physicians could not ascertain a cause of death for 41% of cases, a rate considerably higher than the physicians in our study [15]. This could possibly be due to the greater familiarity of our study's physicians (and also MA) with the early and late neonatal causes of death through their knowledge of the manual and guidelines of Integrated Childhood Management Illness. Such knowledge and awareness of neonatal health issues among both the physicians and the MA would also have been helped by the significant levels of programmatic and research activity in child health ongoing at ICDDR, B.

The MA in our study had a moderate to good agreement with study physician1 and study physician2 for all major categories of death, while for study physician3 this agreement was good for birth asphyxia and fair to moderate for the other causes. Thus, overall, the performance of the MA was consistent with all study physicians. The observed sensitivities and specificities across the five major cause of death varied from 48%-84% and 93%-98%, respectively, between MA and physicians (gold standard). The physicians and the MA took a similar time, around 10-12 minutes for interpreting one VA interview. The symptom-based assessment of causes used in our study was highly culture-specific, and requires a considerable degree of preliminary preparatory work on local perceptions of health and disease. With three years of formal clinical training behind him and the additional orientation provided in verbal autopsy cause assignation, the MA can be expected to have similar cognitive approaches to assigning cause of death as physicians.

Interestingly, agreement between the MA and the physician review method was generally better than the agreement between individual physicians. The rather low inter-rater agreement between the physicians could have been due to individual variations arising from differences in training and work experience.

## Study limitations

The absence of a medically confirmed diagnosis to provide a gold standard for comparing cause of death was a major limitation of the study. In addition, the emphasis on using a single cause of death may have obscured multiple causes contributing to neonatal deaths. Finally, we would like to emphasize that discrepancies between the MA and the physicians do not imply that either assessor was correct, and that our study was not a validation study, rather we looked at the level of agreement between these assessors.

## Conclusion

To achieve the Millennium Development Goal 4, it is important to understand more about the causes of mortality of neonates in the developing world which are an increasing proportion of under-5 child deaths in countries such as Bangladesh. Both resource and practical issues make use of medical autopsy difficult and unlikely to be used for the majority of neonatal deaths as these occur in community settings. However, the limited repertoire of clinical signs and symptoms in most neonatal illnesses and their overlapping features creates difficulty in assigning cause of death with VA, which is often used in developing world community settings as a means of determining cause of death and where medically confirmed gold standard diagnoses are unavailable.

In this study, major causes of neonatal death could be determined by both physicians and the MA trained to interpret VA interviews. Physicians and the MA are generally knowledgeable about the disease profile of a geographical area and can use their clinical judgments and understanding when assigning cause of death. An MA can diagnose a cause of death for all ICD-10 codes by applying their clinical judgment; in contrast, there are no algorithms available for many of the ICD-10 codes. A well-trained MA can therefore be considered as an alternative to physicians for classifying major causes of neonatal death in settings such as rural areas where physicians are scarce, and they will cost much less than a doctor. However, we did not examine the factors affecting the MA's performance, and this could be explored at greater depths in future research. Much larger numbers of trained medical assistants need to be assessed for their capability to do this before their employment in such positions would be justified.

## Competing interests

The authors declare that they have no competing interests.

## Authors' contributions

HRC carried out the study design, data management, analysis and drafted the manuscript. NA manages all the data and PKS manages the Matlab HDSS, and participated in the study design and revising the manuscript. SCT and MA provided help with the study design, data analysis, write-up and editing of the manuscript, while MY contributed to the study by reading the manuscript critically and helping with revisions of the manuscript. All the authors approved the final version of the manuscript.
